# Polyamide Fiber Reinforced Shotcrete for Tunnel Application

**DOI:** 10.3390/ma9030163

**Published:** 2016-03-05

**Authors:** Joong Kyu Jeon, WooSeok Kim, Gyu Yong Kim, Chan Ki Jeon

**Affiliations:** 1 R & BD Center, Kolon Global Corp., Yongin, Gyunggi 17023, Korea; jkjeon31@kolon.com; 2 Department of Civil Engineering, Chungnam National University, Daejeon 34134, Korea; 3 Department of Architectural Engineering, Chungnam National University, Daejeon 34134, Korea; gyuyongkim@cnu.ac.kr; 4 Department of Urban Construction Engineering, Incheon National University, Incheon 22012, Korea; johnland@incheon.ac.kr

**Keywords:** fiber, macro, polyamide, shotcrete, tunnel

## Abstract

This study intends to establish the mechanical properties of polyamide fiber reinforced shotcrete (PAFRS) in terms of compressive and flexural strengths, in accordance with ASTM C1609/C1609M-12. The mechanical properties identified the influence of polyamide fiber content on the PAFRS strength. This study evaluated the toughness of PAFRS and proposed additional toughness level criteria to better represent organic fiber performance. In addition, the fiber rebounding rate and PAFRS performance in tunneling application were evaluated based on a tunnel application in Korea. PAFRS with 0.6%~0.8% volume content in tunneling shotcrete could significantly improve flexural ductility, toughness, and ultimate load capacity. Fiber rebounding tests exhibited a low rebounding rate (8.5%) and low fiber drop (63.5%). Therefore, PAFRS applied to a tunnel exhibited stability and constructability.

## 1. Introduction

Concrete and cementitious materials are vulnerable to tension developing in individual components and structures. Reinforcing materials such as steel fibers [1,2,3,4,5,6] are commonly used in concrete structures to control cracks [7,8,9,10,11,12]. The application of steel fiber reinforced shotcrete (SFRS) in tunneling construction has been part of tunneling practice since the 1970s, especially in Europe and the United States [13,14,15,16]. SFRS is characterized by ductile behavior, namely better post-cracking strength and energy absorption, where the latter is referred to as the “toughness” of a material. More specifically, toughness is the amount of energy that is absorbed before and after fracture [16].

Polyamide fiber reinforced shotcrete (PAFRS) was developed to improve mechanical properties and workability. The detailed manufacturing process has been described in other documents [17,18]. A comparison of 0.47 m diameter and 30 mm long polyamide (PA) fiber to 1.0 mm × 0.5 mm rectangular and 42 mm long polypropylene (PP) fiber [19] shows that 650 MPa tensile strength of PA fiber is higher than 550 MPa of PP fiber, though PA fiber (3 GPa) has less elastic modulus than PP fiber (8.2 GPa). Note also that a higher density of PA fiber (1.14 g/cm^3^) compared to PP fiber (0.9–0.92 g/cm^3^) is advantageous because higher density of PA fiber prevents the fiber from floating in the cement matrix.

Compared to steel fibers, PA fiber has lower weight density, which prevents the fiber from sinking down in the cement matrix and improves the durability without corrosion. Steel fibers may sometimes induce a mixing problem that prevents a uniform distribution of fibers in concrete [20]. The improved mechanical and mixing properties and adhesion characteristics of PA fiber lead to improved workability and less rebounding during spouting of shotcrete.

The objective of this study is to establish PAFRS mechanical properties in terms of compressive and flexural strengths, in accordance with ASTM C1609/C1609M-12 [21]. The mechanical properties reveal the influence of PA fiber content on the PAFRS strength. This study evaluated the toughness of PAFRS using the Morgan level [14] and proposed additional toughness level criteria to better represent organic fiber performance. In addition, based on PAFRS application in Korea, the rebounding rate and performance in a tunneling application were evaluated. PA fibers in tunneling shotcrete significantly improved flexural ductility, toughness, and ultimate load capacity.

## 2. Characterization of PAFRS Flexural Strength

**Figure 1:**
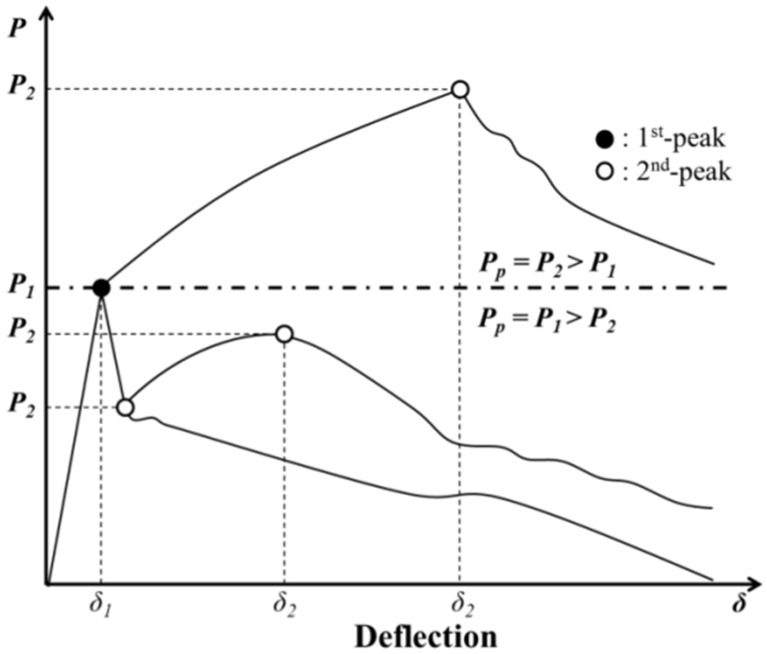
Typical load-deflection responses of FRC.

**Figure 2:**
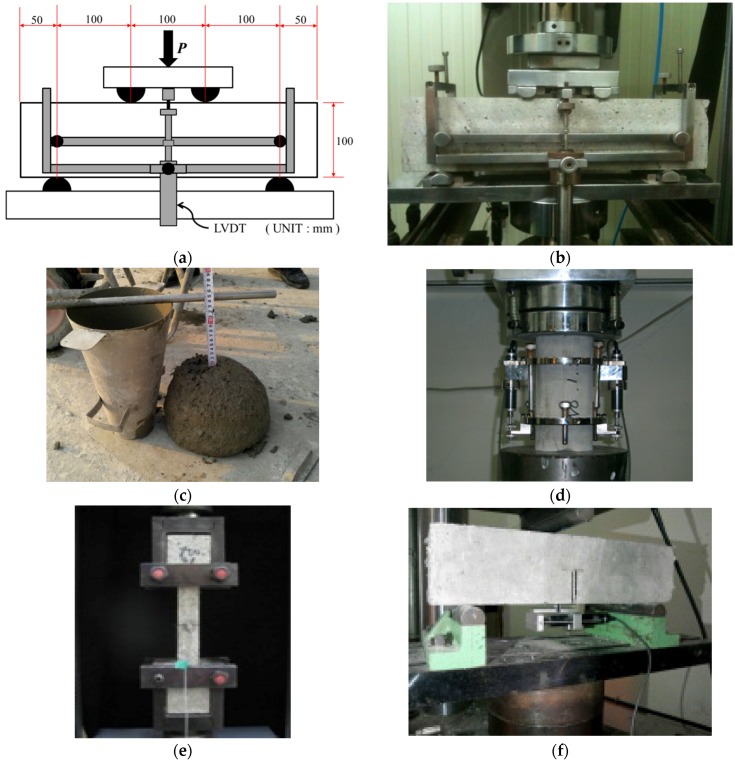
(**a**) Specimen geometry for bending test; (**b**) test set-up picture of bending test; (**c**) slump test; (**d**) compressive test; (**e**) direct tensile test; (**f**) fracture test.

Flexural strength of PAFRSs can be tested in accordance with ASTM C1609/C1609M-12 [21]. As in Figure 1, this study identified two peak loads: (1) the first-peak load ($P_{1}$) on the load-deflection curve, which was caused by crack initiation of the specimen; and (2) the second-peak load ($P_{2}$) on the load-deflection curve when the PA fibers reached the ultimate strength. Corresponding strengths and deflections are denoted as $f_{1}$ and $\delta_{1}$ for $P_{1}$ and $f_{2}$ and $\delta_{2}$ for $P_{2}$, respectively. Residual loads ($P_{600}^{D}$ and $P_{150}^{D}$) and strength ($f_{600}^{D}$ and $f_{150}^{D}$) at a net deflection of *L*/600 and *L*/150 (*L* = clear span) were measured for a beam with a depth of *d* (=100 mm in Figure 2a). Toughness ($T_{150}^{D}$) and equivalent flexural strength ratio ($R_{T,150}^{D}$) at a net deflection of *L*/150 (=300 mm in Figure 2a) were also identified. Flexural strength of $f_{1}$ and $f_{2}$, equivalent bending strength of $f_{e}$ and equivalent flexural strength ratio of $R_{T,150}^{D}$ were computed as follows:

(1) $$f_{i}=\frac{P_{i}L}{bd^{2}}$$

(2) $$f_{e}=\frac{A_{b}L}{\delta_{150}bd^{2}}$$

(3) $$R_{T,150}^{D}=\frac{f_{i}}{f_{e}}\times 100\;(\%)=\frac{150\cdot T_{150}^{D}}{f_{1}\cdot b\cdot d^{2}}\times 100\;(\%)$$

where $P_{i}$ = *i*^th^ peak load at $\delta_{i}$; *L* = clear span length (=300 mm); *b* and *d* = beam cross-section width and depth at the fracture surface, respectively; $A_{b}$ = area under the load-deflection curves up to $\delta_{150}$ (N·mm); and $\delta_{150}$ = deflection of *L*/150 (=2.0 mm).

Based on the deflection ($\delta_{1}$) at the first-peak load, three additional points were investigated at 3$\delta_{1}$, 5.5$\delta_{1}$, and 10.5$\delta_{1}$ as per ASTM C1018-97 [22]. The current ASTM standard [21] specifies $\delta_{1}$, $\delta_{p}$ (deflection at the peak load regardless of the first-peak or second-peak), L/600 and L/150. However, PAFRS occasionally exhibits a larger second peak than the first peak depending on the PA fiber volume content. Thus, this study clearly stipulated pre-(1st peak) and post(2nd peak)-cracking strengths. Also, PAFRS displays yielding after cracking before the PA fiber reaches its ultimate strength, similar to hyper-elastic materials. Thus, additional points at 3$\delta_{1}$, 5.5$\delta_{1}$, and 10.5$\delta_{1}$ were used in this study.

## 3. PA Fiber Volume Content Influence on PAFRS Mechanical Properties

The PAFRS specimens were prepared with varying PA fiber content ratios from 0.0% to 1.5%. Detailed shotcrete mix design of water to cement ratio (W/C), sand to aggregate ratio (S/a), water (W), cement (C), fly ash (FA), sand (S), gravel (G), and water-reducing admixture (AD) is presented in Table 1. The PAFRS was tested for slump, bending strength, direct tensile strength, and fracture energy, as shown in Figure 2. A bending test was performed in accordance with ASTM C1609/C1609M-12 [21]. A rectangular beam was saw-cut to dimensions of 100 mm × 100 mm × 400 mm. To carry out compressive tests, a φ 100 mm × 200 mm cylindrical specimen was used. A direct tensile test was conducted used a dog-bone shaped specimen. For a fracture energy test, a rectangular specimen of 100 mm × 100 mm × 400 mm, which is the same as to the specimen employed in the bending strength test, was used and the test procedure followed RILEM TCS [23]. The obtained test results with respect to varying PA fiber contents are tabulated in Table 2.

### 3.1. Compressive Strength and Elastic Modulus

**Figure 3:**
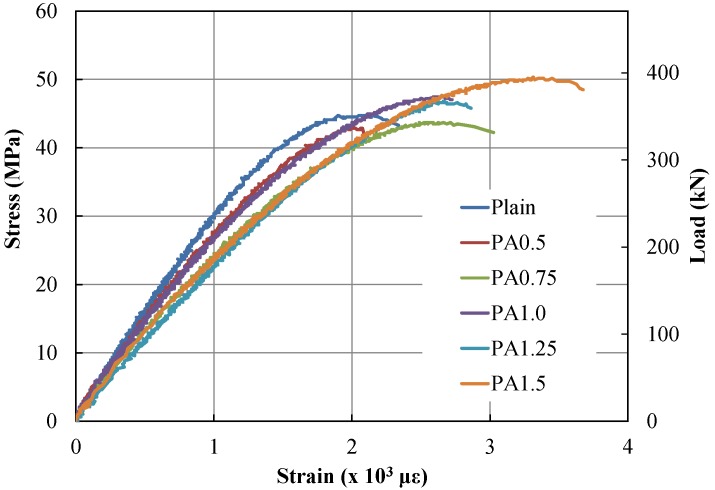
Compressive stress–strain curves of PAFRSs with varying PA contents.

As presented in Figure 3, compressive strength tests were performed for PAFRS specimens with varying PA fiber content from 0.0% to 1.5%. It was clearly observed that the slope or elastic modulus of PAFRS decreased as the PA fiber content increased. Obtained maximum compressive strengths with respect to PA fiber content from 0.0% to 1.5% are shown in Table 2. However, the maximum compressive strengths of PA-0.5 and PA-0.75 (42.86 and 43.69 MPa) were less than that of Plain (44.94 MPa), but those of PA-1.00, PA-1.25 and PA-1.50 (47.44, 49.82 and 50.35 MPa) were larger than that of Plain. This phenomenon is commonly encountered in fiber-reinforced concrete.

The elastic moduli of the PAFRS specimens slightly decreased compared to those of the plain specimen. The elastic modulus of the plain shotcrete mix was larger than those of PAFRSs. The elastic moduli of PAFRSs were not proportional to the PA fiber content. The order of magnitude of the PAFRS elastic modulus was 0.5% > 1.0% > 0.75% >1.5% > 1.25% in terms of PA fiber content.

### 3.2. Direct Tensile Strength Test

**Figure 4:**
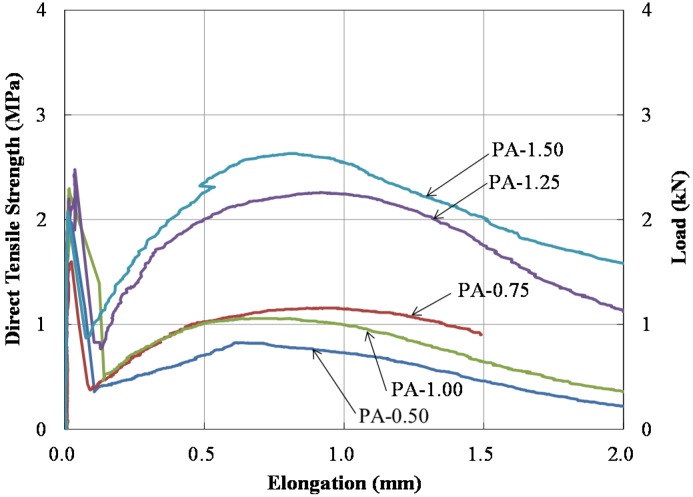
Direct tensile test results.

Direct tensile strength test results are presented in Figure 4. It was observed that the shotcrete toughness was significantly improved by inclusion of PA fibers. The tensile strength tended to linearly increase as the PA fiber content increased, except for the PA-0.75 specimen. The maximum tensile strength is shown in Table 2. In Figure 4, the 1st peak tensile strengths were 2.00, 1.60, 2.48, 2.30, and 2.08 MPa for PA-0.5, 0.75, 1.25, 1.0, and 1.5. The 2nd peak tensile strengths were 0.82, 1.16, 2.26, 0.73, and 2.63 MPa for PA-0.5, 0.75, 1.25, 1.0, and 1.5. All specimens exhibited larger first peak tensile strengths than the second peak strengths except for the PA-1.5 specimen. The PA-1.5 specimen exhibited a larger second peak tensile strength than the first peak tensile strength. It was expected that the PA fiber content generally increased the second peak strength.

### 3.3. Flexural Bending Test Results

**Figure 5:**
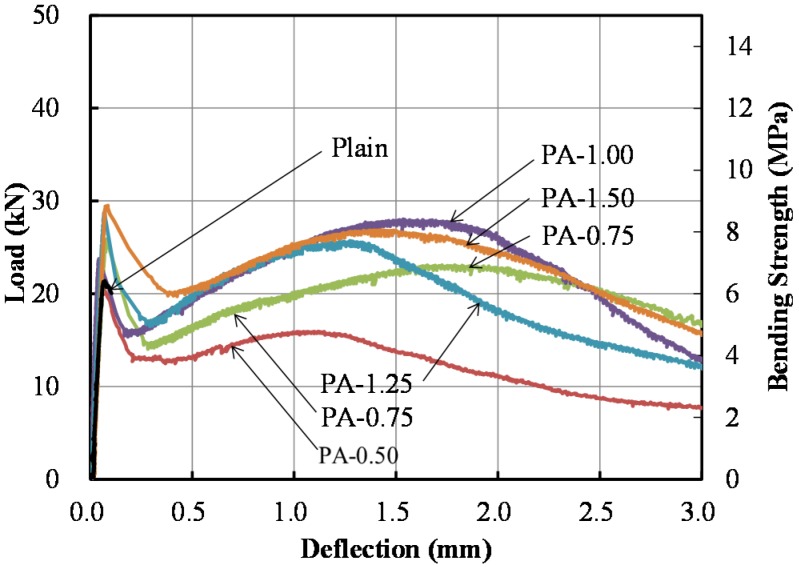
Load-deflection curves of flexural bending tests.

Flexural bending test results are presented in Figure 5. Similar to the direct tensile test results, the flexural bending strengths were significantly improved by PA fibers. As aforementioned, the PAFRS exhibited two distinct peaks: (1) the first peak was induced by the initiation of the crack; and (2) the second peak was reached as the fiber reached its ultimate strength.

**Figure 6:**
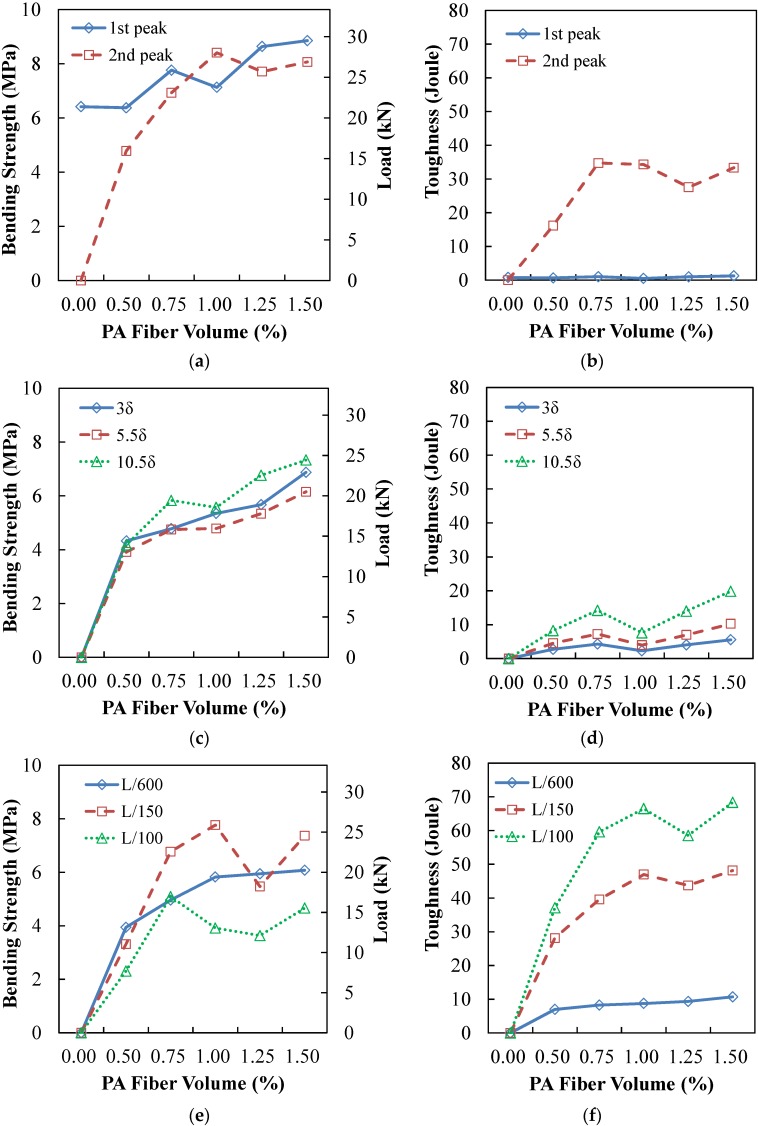
**Figure 6**. (**a**) Bending strength (first and second peaks); (**b**) toughness (first and second peaks); (**c**) bending strength (3δ, 5.5δ, 10.5δ); (**d**) toughness (3δ, 5.5δ, 10.5δ); (**e**) bending strength (L/600, L/150, L/100); (**f**) toughness (L/600, L/150, L/100).

Detailed flexural responses at various points are presented in Figure 6 and Table 3. As shown in Figure 6, it was clearly observed that the bending strength and toughness increased as the PA fiber content increased. Also, the second peak tended to increase as the PA fiber content increased.

## 4. Field and Laboratory Fabricated PAFRS

**Figure 7:**
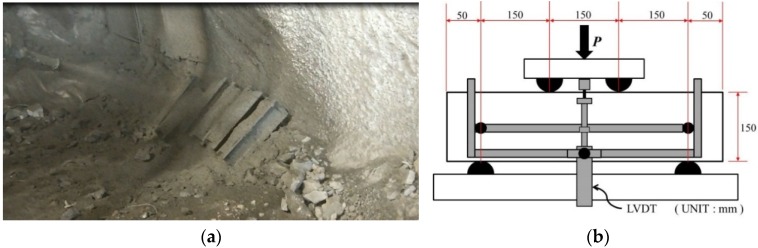
(**a**) Shotcrete specimen fabrication; (**b**) specimen geometry and test setup.

For tunnel application of PAFRS, field and laboratory specimens were fabricated according to the approach discussed earlier, and the flexural performance was evaluated in accordance with ASTM C1609/C1609M-12 [21] and the Morgan level [14]. First, appropriate PA fiber content was determined based on the bending strength in Equation (1) and equivalent bending strength in Equation (2), since compressive strengths of PAFRS in Figure 3 were far higher than the minimum limit in Table 4. There are four applicable Korean tunnel design guidelines for fiber-reinforced shotcrete, listed in Table 4. All four design guidelines specify the minimum compressive strength, bending strength, and equivalent bending strength. For this study, 0.6%~0.8% of PA fiber content was determined to be the optimal content based on Table 3, and both laboratory and field specimens were then prepared. Shotcrete mix design such as the maximum gravel size (G_max_), ratio of water to cement (W/C) and sand to aggregate (S/a), unit weight of water (W), cement (C), crushed sand (CS), gravel (G), polyamide fiber (PA). and water-reducing admixture (AD) is presented in Table 5. The test specimens were fabricated from tunnel lining shotcrete during construction as in Figure 7a and dimensions of specimens and the test setup were slightly modified to meet the Korean standard and are presented in Figure 7b. For each shotcrete mix design, three specimens were prepared and tested.

**Figure 8:**
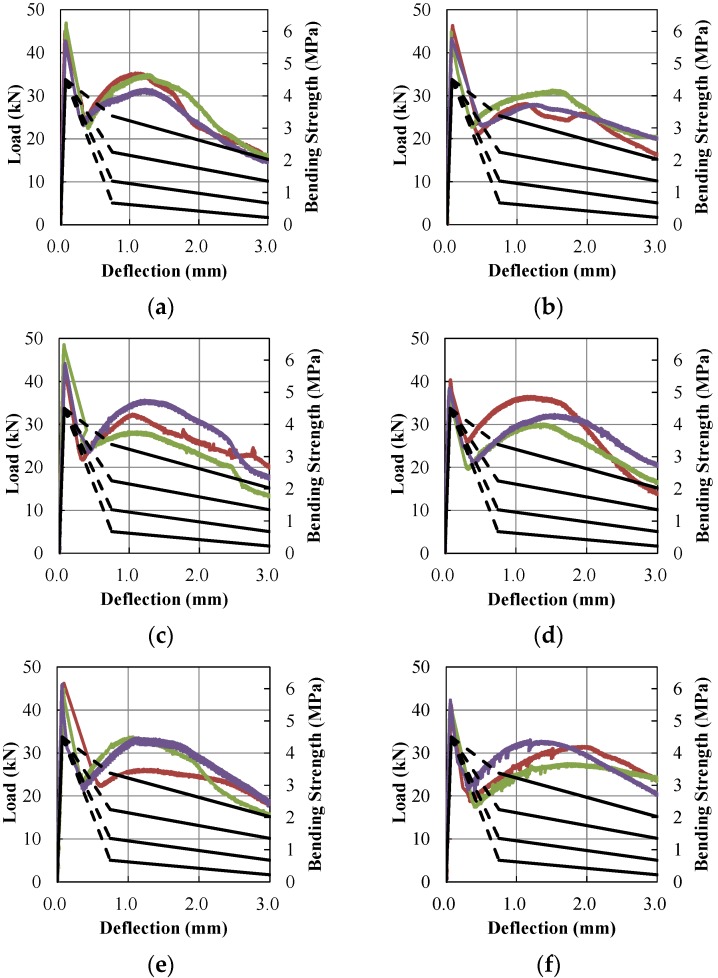
(**a**) PA-0.6L; (**b**) PA-0.6F; (**c**) PA-0.7L; (**d**) PA-0.7F; (**e**) PA-0.8L; (**f**) PA-0.8F.

**Figure 9:**
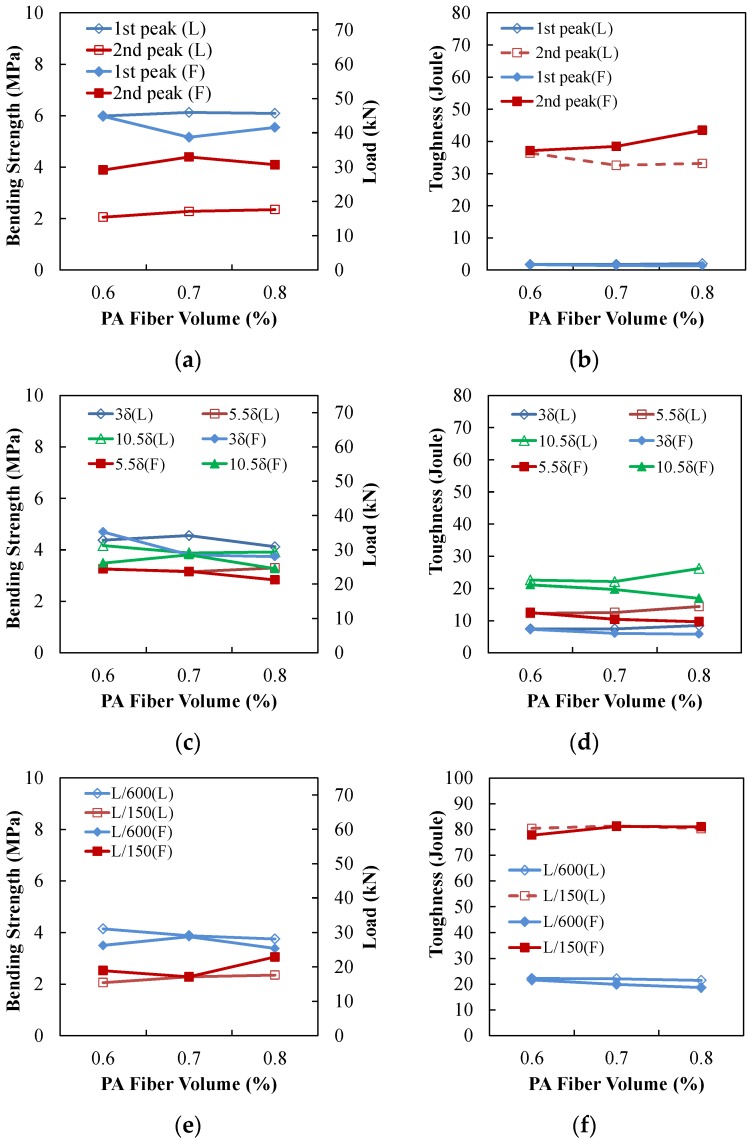
(**a**) Bending strength (first and second peaks); (**b**) toughness (first and second peaks); (**c**) bending strength (3δ, 5.5δ, 10.5δ); (**d**) toughness (3δ, 5.5δ, 10.5δ); (**e**) bending strength (L/600, L/150); (**f**) toughness (L/600, L/150).

Bending and equivalent bending strength test results from the laboratory and field are presented in Table 6. All PAFRS specimens fabricated in both laboratory and field exhibited higher bending and equivalent bending strengths (see Table 7) than the minimum limits in Table 4. Load-deflection relationship of all specimens was also investigated, as in Figure 8. The load-deflection curves of PAFRS specimens were similar to each other due to the small variation of PA fiber content. Detailed PAFRS bending strength and toughness with respect to varying fiber content are presented in Figure 9. The average flexural responses at δ*_1_*, 3δ*_1_*, 5.5δ*_1_*, *L*/600, 10.5δ*_1_*, and L/150 are summarized in Table 7. As shown in the results, the quality and performance of field specimens were similar to those of laboratory specimens.

Based on the load-deflection curves in Figure 8, Morgan levels [14] for each specimens were determined to identify the toughness performance level, as presented in Table 8. The Morgan level evaluates the strength of steel fiber reinforced shotcrete at $\delta_{1}$ *L*/600 and *L/*150. However, PAFRSs exhibit different load-deflection history compared to SFRCs, although the Morgan level was originally suggested for SFRCs. After the first peak in Figure 8, the load-deflection curve drops until the PA fiber takes the load, then starts to increase up to the second peak or ultimate fiber capacity. Figure 9a shows that the second peaks of all specimens were larger than or equal to the first peak. As shown in Figure 8, some of the load-deflection curves of PAFRS specimens at *L*/600 after the first peak dropped below the Morgan level IV. However, the load-deflection curves increase again, beyond level IV. Thus, this study evaluated the specimen capacity in terms of toughness. The toughness of each specimen was compared to the toughness specified by Morgan load-deflection curves. The toughness Morgan levels [14] of all specimens were Level IV while the strength Morgan levels of all specimens were Level III.

## 5. PAFRS Shotcrete Application Results

**Figure 10:**
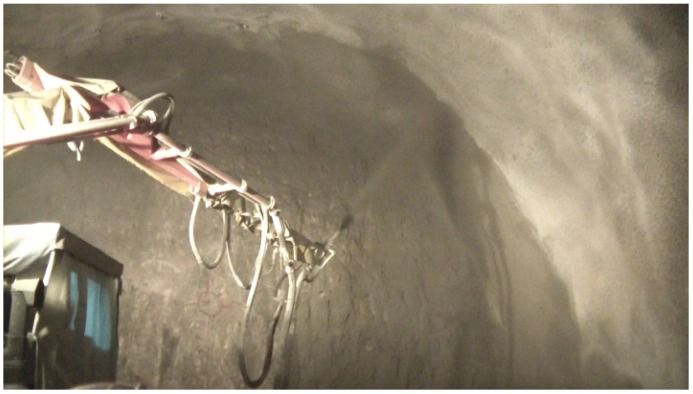
Shotcrete spouting and rebounding test.

The PAFRS of PA-0.7F in Table 5, Table 6 and Table 7 was conservatively selected and was used in tunnel shotcrete to evaluate shotcrete stresses and rebounding rate for constructability, as presented in Figure 10. For comparison, steel fiber (0.5 mm diameter, 30 mm long and hooked type) reinforced shotcrete was also applied at the same construction site. Also, four sample cores with an average thickness of 168 mm were collected to confirm the shotcrete thickness and contact to the rock. The flexural strength of the specimen was satisfactory compared to the tunnel codes, as presented in Table 7.

Also, the flexural stress limits by Korean Tunnel Design Specifications [27] were checked. The measured shotcrete stresses are presented in Table 9. Both PA and steel fiber reinforced shotcrete satisfied the allowable limits (allowable flexural compressive stress = 8.4 MPa, allowable flexural tensile stress = 0.60 MPa). The maximum compressive and tensile stresses of PA shotcrete were observed at the crown and the East springline, respectively, but the stress was only 2.5% and 10% with respect to the allowable stresses.

**Figure 11:**
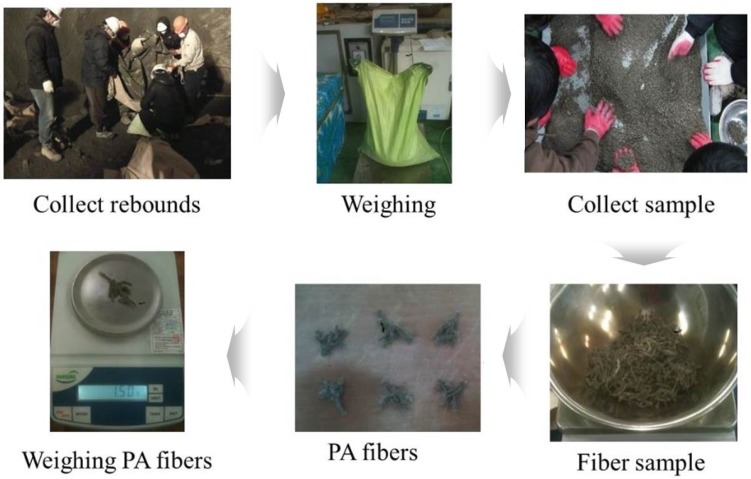
PA shotcrete rebounding test procedure.

Finally, PA shotcrete rebounding was checked to identify the fiber ratio included and dropped in the final shotcrete. The test procedure is presented in Figure 11. The rebounding test results are presented in Table 10. The average rebounding rate was 8.5%, which is less than the limit of 12.5% designated by Korean Tunnel Design Specifications [27]. The average dropped fiber rate was 63.5%. The average fiber content rate was 103.4%.

## 6. Conclusions

This study performed laboratory tests to investigate the influence of PA fiber content and to determine the appropriate PA fiber content for tunnel shotcrete application. As expected, PAFRS flexural performance improved as PA fiber contents increased from 0.5% to 1.5%. Since the experimental results of all specimens exhibited far higher flexural performance than the required levels, 0.6~0.8% of PA fiber content was selected for a field application. In this study, three PAFRS specimens for each PA fiber content were prepared in the laboratory and field to compare the flexural performance and toughness. Finally, PA-0.7 was selected and used in tunnel shotcrete. In the field, steel fiber reinforced shotcrete was also used for comparison. Both PA and steel fiber reinforced shotcrete satisfied the allowable stress limits by Korean Tunnel Design Specifications [27]. Also, fiber rebounding tests exhibited the constructability and quality of PA shotcrete, based on its low rebounding rate (8.5%) and low fiber drop (63.5%). Based on these results, the fiber content in the shotcrete was expected to be 103.4% Therefore, PA shotcrete applied in a tunnel exhibited stability and constructability.

## Figures and Tables

**Table 1:** Shotcrete mix design.

Types	Fiber Content (vol %)	W/C (%)	S/a (%)	Unit Weight (kg/m^3^)					AD (B * %)
				W	C	FA	S	G	
Plain	-	40	44.4	163	326	82	749	938	0.3
PA-0.50	0.50	40	44.4	163	326	82	749	938	1.0
PA-0.75	0.75	40	55.0	180	360	90	904	740	1.0
PA-1.00	1.00	40	55.0	180	360	90	904	740	1.2
PA-1.25	1.25	40	55.0	188	376	94	883	722	1.6
PA-1.50	1.50	40	55.0	188	418	104	857	701	1.7

* weight percentage to binder.

**Table 2:** PAFRS mechanical properties.

Specimen	Slump (mm)	Compressive Strength (MPa)	Elastic Modulus (MPa)	Direct Tensile Test (MPa)	Bending Strength (MPa)	Equivalent Bending Strength (MPa)	Fracture Energy (N·m/m^2^)
Plain	150	44.94	30,119	-	6.42	-	908.45
PA-0.50	155	42.86	28,930	2.00	6.38	4.22	4010.66
PA-0.75	140	43.69	25,784	1.60	7.76	5.94	5674.95
PA-1.00	130	47.44	28,783	2.30	7.13	7.05	7286.54
PA-1.25	140	49.82	23,194	2.48	8.64	6.57	8750.07
PA-1.50	130	50.35	23,357	2.63	8.85	7.22	10,132.19

**Table 3:** Average value of flexural responses of PAFRS.

Specimen		First-Peak							3δ*_1_*								5.5δ*_1_*						10.5δ*_1_*						
		*P_1_*(N)		δ*_1_* (mm)	*f_1_* (MPa)		*T_1_* (Joule)		*P_3_*_δ_**(N)		δ*_3_*_δ_ (mm)		*f_3_*_δ_ (MPa)		*T_3_*_δ_ (Joule)		*P_5.5_*_δ_**(N)		δ*_5.5_*_δ_ (mm)	*f_5.5_*_δ_**(MPa)	*T_5.5_*_δ_**(Joule)		*P_10.5_*_δ_**(N)		δ*_10.5_*_δ_ (mm)		*f_10.5_*_δ_**(MPa)		*T_10.5_*_δ_**(Joule)
Plain		21,391		0.072	6.42		0.793		-		0.215		-		-		-		0.394	-	-		-		0.751		-		-
PA-0.50		21,251		0.057	6.38		0.664		14,415		0.170		4.32		2.735		13,069		0.312	3.92	4.547		13,594		0.595		4.19		8.275
PA-0.75		25,872		0.079	7.76		1.028		15,918		0.237		4.78		4.281		15,817		0.435	4.75	7.221		19,429		0.830		5.83		14.231
PA-1.00		23,769		0.042	7.13		0.514		17,824		0.126		5.35		2.297		15,952		0.230	4.79	3.973		18,566		0.440		5.57		7.581
PA-1.25		28,789		0.069	8.64		0.992		18,908		0.206		5.67		4.040		17,783		0.377	5.34	6.993		22,545		0.720		6.76		14.020
PA-1.50		29,506		0.087	8.85		1.285		22,914		0.260		6.87		5.550		20,503		0.476	6.15	10.257		24,445		0.909		7.33		19.860
**Specimen**	**Second-Peak**									***L*** **/600 (=0.5 mm)**						***L*** **/150 (=2.00 mm)**								**L/100 (=3.00 mm)**					
	***P_2_* (N)**		**δ*_2_* (mm)**			***f_2_* (MPa)**		***T_2_* (Joule)**		**$P_{600}^{100}$ (N)**		**$f_{600}^{100}$ (MPa)**		**$T_{600}$ (Joule)**		**$P_{150}^{100}$ (N)**		**$f_{150}^{100}$ (MPa)**		**$T_{150}$ (Joule)**		**$R_{T,150}^{100}$ (%)**		**$P_{100}^{100}$ (N)**		**$f_{100}^{100}$ (MPa)**		**$T_{100}$ (Joule)**	
Plain	-		-			-		-		-		-		-		-		-		-		-		-		-		-	
PA-0.50	15,928		1.117			4.78		16.180		13,129		3.94		6.975		11,035		3.31		28.126		66.2		7686		2.31		37.100	
PA-0.75	23,102		1.788			6.93		34.725		16,534		4.96		8.276		22,564		6.77		39.575		76.5		16,959		5.09		59.614	
PA-1.00	28,022		1.535			8.41		34.313		19,409		5.82		8.738		25,887		7.77		46.979		98.8		13,045		3.91		66.477	
PA-1.25	25,697		1.273			7.71		27.564		19,805		5.94		9.342		18,226		5.47		43.712		75.9		12,113		3.63		58.539	
PA-1.50	26,886		1.428			8.07		33.347		20,246		6.07		10.683		24,553		7.37		48.141		81.6		15,527		4.66		68.313	

**Table 4:** Korean design guidelines for tunnel shotcrete.

Property	Unit	Korean Highway Design Specifications (2012) [24]	Korean Railroad Design Specifications (2011) [25]	Korean High Speed Railroad Design Specifications (2005) [26]	Korean Tunnel Design Specifications (2007) [27]
Compressive Strength	MPa	>10 (1 day)	>10 (1 day)	>10 (1 day)	>10 (1 day)
		>21 (28 days)	>21 (28 days)	>21 (28 days)	>21 (28 days)
Bending Strength	MPa	>4.4 (28 days)	>4.5 (28 days)	>4.5 (28 days)	>4.5 (28 days)
Equivalent Bending Strength	MPa	-	>3.0 (28 days)	>3.0 (28 days)	>3.0 (28 days)

**Table 5:** Shotcrete mix design.

Specimen	G_max_ (mm)	W/C (%)	S/a (%)	Unit Weight (kg/m^3^)						AD (B *** %)
				W	C	S	CS	G	PA	
PA-0.6L *	10	43.5	60	211	485	472	463	617	6.84	0.9
PA-0.6F **	10	43.5	60	211	485	472	463	617	6.84	1.0
PA-0.7L	10	43.5	60	211	485	472	463	617	7.98	1.0
PA-0.7F	10	43.5	60	211	485	472	463	617	7.98	1.1
PA-0.8L	10	43.8	60	211	482	385	578	651	9.12	1.1
PA-0.8F	10	43.8	60	211	482	385	578	651	9.12	1.2

* PA-xxL: mixed in laboratory; ** PA-xxF: mixed in field; *** weight percentage to binder.

**Table 6:** Bending and equivalent bending strength.

Specimen	Bending Strength (MPa)				Equivalent Bending Strength (MPa)			
	SP-1	SP-2	SP-3	Avg.	SP-1	SP-2	SP-3	Avg.
PA-0.6L	6.01	6.25	5.70	5.99	3.63	3.72	3.38	3.57
PA-0.6F	6.18	5.97	5.78	5.98	3.33	3.59	3.46	3.46
PA-0.7L	6.02	6.48	5.89	6.13	3.60	3.32	3.93	3.62
PA-0.7F	5.39	4.97	5.13	5.16	3.83	3.32	3.67	3.61
PA-0.8L	6.16	5.98	6.13	6.09	3.41	3.58	3.74	3.57
PA-0.8F	5.49	5.50	5.65	5.55	3.63	3.42	3.76	3.60

PA-xxL: mixed in laboratory; PA-xxF: mixed in field.

**Table 7:** Average value of flexural responses of PAFRS.

**Specimen**	**First Peak**								**3δ*_1_***								**5.5δ*_1_***							
	***P_1_* (N)**		**δ*_1_* (mm)**		***f_1_* (MPa)**		***T_1_* (Joule)**		***P_3_*_δ_** (N)**		**δ*_3_*_δ_ (mm)**		***f_3_*_δ_ (MPa)**		***T_3_*_δ_ (Joule)**		***P_5.5_*_δ_** (N)**		**δ*_5.5_*_δ_ (mm)**		***f_5.5_*_δ_** (MPa)**		***T_5.5_*_δ_** (Joule)**	
PA-0.6L	44,921		0.073		5.99		1.757		32,854		0.218		4.38		7.382		24,467		0.399		3.26		12.275	
PA-0.6F	44,816		0.070		5.98		1.710		35,242		0.210		4.70		7.275		24,408		0.385		3.25		12.522	
PA-0.7L	45,985		0.071		6.13		1.753		34,171		0.214		4.56		7.428		23,599		0.392		3.15		12.532	
PA-0.7F	38,719		0.070		5.16		1.411		28,551		0.210		3.81		6.065		23,710		0.384		3.16		10.424	
PA-0.8L	45,685		0.087		6.09		2.027		30,923		0.262		4.12		8.493		24,743		0.481		3.30		14.365	
PA-0.8F	41,598		0.065		5.55		1.359		28,009		0.195		3.73		5.811		21,249		0.357		2.83		9.681	
**Specimen**	***L*/600 (=0.75 mm)**					**10.5δ*_1_***							**Second Peak**						***L*/150 (=3.00 mm)**					
	**$P_{600}^{100}$ (N)**	**$f_{600}^{100}$ (MPa)**		**$T_{600}$ (Joule)**		***P_10.5_*_δ_** (N)**		**δ*_10.5_*_δ_ (mm)**		***f_10.5_*_δ_** (MPa)**		***T_10.5_*_δ_** (Joule)**	***P_2_* (N)**	**δ*_2_* (mm)**		***f_2_* (MPa)**		***T_2_* (Joule)**	**$P_{150}^{100}$ (N)**	**$f_{150}^{100}$ (MPa)**		**$T_{150}$ (Joule)**		**$R_{T,150}^{100}$ (%)**
PA-0.6L	31,106	4.15		22.194		31,266		0.763		4.17		22.622	33,928	1.187		4.52		36.389	15,444	2.06		80.423		59.7
PA-0.6F	26,294	3.51		21.551		26,171		0.735		3.49		21.144	29,159	1.301		3.89		37.141	18,929	2.52		77.813		57.9
PA-0.7L	29,118	3.88		22.041		29,120		0.748		3.88		22.151	32,098	1.084		4.28		32.564	17,093	2.28		81.402		59.3
PA-0.7F	28,809	3.84		19.915		28,582		0.734		3.81		19.711	32,983	1.351		4.40		38.458	17,129	2.28		81.163		69.8
PA-0.8L	28,157	3.75		21.431		29,421		0.918		3.92		26.217	31,179	1.148		4.16		33.159	17,623	2.35		80.382		58.7
PA-0.8F	25,391	3.39		18.639		24,518		0.682		3.27		16.936	30,708	1.629		4.09		43.474	22,904	3.05		81.039		64.9

**Table 8:** Morgan level based on strength and toughness.

Method Basis	Specimen No.	PA-0.6L	PA-0.6F	PA-0.7L	PA-0.7F	PA-0.8L	PA-0.8F
Strength	1	IV	IV	IV	III	III	III
	2	IV	IV	III	IV	IV	III
	3	III	III	IV	IV	IV	IV
	Min. Level	III	III	III	III	III	III
Toughness	1	IV	IV	IV	IV	IV	IV
	2	IV	IV	IV	IV	IV	IV
	3	IV	IV	IV	IV	IV	IV
	Min. Level	IV	IV	IV	IV	IV	IV

**Table 9:** Measured shotcrete stresses.

Location	Springline (East)		Springline (West)		Crown	
Fiber	Max. Flexural Compressive Stress (MPa)	Max. Flexural Tensile Stress (MPa)	Max. Flexural Compressive Stress (MPa)	Max. Flexural Tensile Stress (MPa)	Max. Flexural Compressive Stress (MPa)	Max. Flexural Tensile Stress (MPa)
PA	-	0.06	0.19	0.06	0.21	0.05
Steel	-	0.37	0.01	0.23	0.36	0.27

**Table 10:** PAFRS rebounding test results.

Field Test	Spouting Volume (m^3^)	Spouting Weight (kg)	Fiber Content (kg/m^3^)	Rebounded Weight (kg)	Rebounded Rate (%)	Fiber Content * (%)	Dropped Fiber ** (%)
1st	0.3	682.2	8.00	59.4	8.71	103.55	62.83
2nd	1.0	2274.0	8.00	188.8	8.30	103.24	64.23

* $\text{Fiber Content }(\%)=\frac{\text{Theoretical Number of Fibers in Shotcrete}}{\text{Actual Number of Fibers in Shotcrete}}\times 100$; ** $\text{Dropped Fiber }(\%)=\frac{\text{Theoretical Number of Fibers in Rebounded}}{\text{Actual Number of Fibers in Rebounded}}\times 100$
